# The Impacts of COVID-19 Lockdowns on Road Transport Air Pollution in London: A State-Space Modelling Approach

**DOI:** 10.3390/ijerph21091153

**Published:** 2024-08-30

**Authors:** Hajar Hajmohammadi, Hamid Salehi

**Affiliations:** 1 Centre for Primary Care, Wolfson Institute of Population Health, Queen Mary University of London, London E1 4NS, UK; h.hajmohammadi@qmul.ac.uk; 2 School of Engineering, University of Greenwich, Chatham ME4 4TB, UK

**Keywords:** COVID-19 lockdowns, air pollution, state-space modelling, fine particles

## Abstract

The emergence of the COVID-19 pandemic in 2020 led to the implementation of legal restrictions on individual activities, significantly impacting traffic and air pollution levels in urban areas. This study employs a state-space intervention method to investigate the effects of three major COVID-19 lockdowns in March 2020, November 2020, and January 2021 on London’s air quality. Data were collected from 20 monitoring stations across London (central, ultra-low emission zone, and greater London), with daily measurements of NO_x_, PM_10_, and PM_2.5_ for four years (January 2019–December 2022). Furthermore, the developed model was adjusted for seasonal effects, ambient temperature, and relative humidity. This study found significant reductions in the NO_x_ levels during the first lockdown: 49% in central London, 33% in the ultra-low emission zone (ULEZ), and 37% in greater London. Although reductions in NO_x_ were also observed during the second and third lockdowns, they were less than the first lockdown. In contrast, PM_10_ and PM_2.5_ increased by 12% and 1%, respectively, during the first lockdown, possibly due to higher residential energy consumption. However, during the second lockdown, PM_10_ and PM_2.5_ levels decreased by 11% and 13%, respectively, and remained unchanged during the third lockdown. These findings highlight the complex dynamics of urban air quality and underscore the need for targeted interventions to address specific pollution sources, particularly those related to road transport. The study provides valuable insights into the effectiveness of lockdown measures and informs future air quality management strategies.

## 1. Introduction

Air pollution has emerged as a major concern for urban and suburban areas around the world, having negative effects on both human health [1,2], anthropogenic ecosystems [3], and climate [4]. In cities such as London, a major source of air pollution is road transport, which produces nearly half of all nitrogen oxides (NO_x_) and particulate matter (PM_10_ and PM_2.5_) into the air [5]. Air quality in London has improved in recent years as a result of restrictive policies, such as ultra-low emission [6]. Despite this progress, air pollution levels in London will continue to exceed WHO clean air guidelines in 2025 and 2030 [7]. 

The emergence of the COVID-19 pandemic in 2020 resulted in the introduction of legal restrictions, known as lockdowns, on individual activities. Governments around the world implemented extraordinary measures in 2020, including restrictions on road and air travel and restricted human movement. These lockdowns encompassed the extensive cessation of diverse activities, including limitations on the movement of vehicles, temporary cessation of industrial and productive operations, closures of non-essential establishments, offices, and educational institutions. The impacts of these lockdowns were examined by several studies in different locations, such as China [8,9,10,11], Brazil [12], central Europe [13], and India [14,15].

Some studies have compared the levels of atmospheric contaminants observed during periods of lockdown to those recorded in the same year prior to or after the time of lockdown [16,17]. Other research examined the same time period three years prior to the onset of the pandemic [18], also frequently confining their analysis to singular pollutants.

During the lockdown, numerous studies reported lower levels of particulate matter (PM_10_ and PM_2.5_), nitrogen dioxide (NO_2_), carbon monoxide (CO), Sulphur dioxide (SO_2_), and ozone (O_3_) when compared to pre-lockdown situations and/or prior years [18,19,20,21]. In some other studies, however, the levels of PM_2.5_ (particularly secondary organic aerosols) were reported to stay constant or increase as a result of volatile organic compounds (VOCs) [22,23]. Furthermore, the concentrations of O_3_, which is a type of air pollution formed as a result of chemical reactions, have often been seen to remain constant or even rise [24]. This inconsistency can be attributed to variations in emission levels from specific sources, the proximity of sources to air quality monitoring stations, prevailing meteorological conditions, chemical reactions involving atmospheric oxidants, and the efficiency of pollutant removal through atmospheric deposition.

Air pollutants are emitted from several sources, including factories, on-road vehicles, ships, and airplanes, as well as from natural sources such as biomass burning, dust storms, and seas. Lockdown measures greatly limited urban mobility in many areas, significantly reducing transportation-related air pollution. The emission characteristics of traffic-related pollutants depend on both the number of vehicles on the road and the composition of traffic, including motorbikes, passenger cars, buses, utility vehicles, and heavy-duty trucks [25].

London has a complex and dense transportation network; hence, the impact of reduced traffic during lockdowns presents a unique opportunity to study changes in air quality. The lockdown period provided an unplanned but valuable scenario to observe the effects of drastic reductions in traffic and industrial activities on air pollution levels. Furthermore, understanding the specific changes in air quality due to lockdown measures can inform future urban planning and public health policies. If certain reductions in pollutants are observed during these periods, this may suggest potential strategies for reducing air pollution under normal circumstances. This could include promoting remote work, increasing investments in public transportation, and encouraging the use of cleaner vehicle technologies.

In this study, we used a state-space intervention method to explore the effects of the multiple COVID-19 restrictions on London’s air quality. State-space modelling is an advanced time series model which can investigate both temporal and spatial changes in the air pollution levels (daily measurement at monitoring stations), and it can incorporate several interventions (such as COVID-19 lockdowns) [6,26,27]. The state-space intervention method is particularly advantageous for this kind of study because it can simultaneously account for the time-varying nature of air pollution data and the multiple interventions (i.e., different stages of lockdown measures). It allows for a more comprehensive understanding of the temporal dynamics and the impact of multiple factors on air quality. We used an extensive dataset including daily air pollution measurements of four years (2019–2022) from 20 monitoring stations across London, which facilitated the investigation of distinct trends and patterns in London’s air pollution levels over time and location. Meteorological data, including ambient temperature and relative humidity, were also considered in this model.

While various studies have explored the effects of COVID-19 lockdowns on air pollution using more traditional statistical approaches, such as regression analysis and the difference-in-differences method, the state-space intervention method offers a unique and advanced approach that has not yet been widely applied to this specific context in London. By utilizing this method, our study aims to fill this gap and provide more detailed and actionable insights into the impacts of lockdown measures on urban air quality. This approach not only enhances our understanding of the immediate effects of COVID-19 restrictions but also contributes to the broader field of environmental health research by demonstrating the utility of state-space models in analysing complex, real-world data.

## 2. Study Design

**Figure 1:**
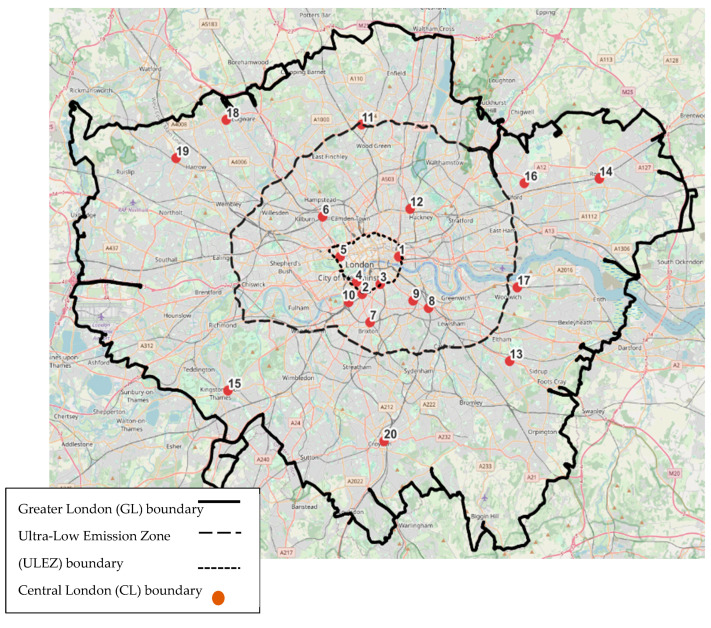
Locations of the air pollution monitoring stations across London.

For this study, a total of 20 air quality monitoring stations across London (Figure 1) were selected to analyse the impacts of COVID-19 lockdowns on air pollution. The air quality data were collected for a four-year period (2019–2022) to capture pre-lockdown, lockdown, and post-lockdown phases from the London Air Quality Network (LAQN) [28]. This dataset includes daily measurements of key pollutants including NO_2_, PM_10_, and PM_2.5_. The selected stations were strategically distributed to capture variations across different areas with distinct traffic patterns and emission characteristics:

Central London: Five stations were located in central London, an area characterized by high traffic density, significant commercial activity, and stringent emission regulations. This area represents the core urban environment where the impact of lockdowns on traffic-related emissions is expected to be most pronounced.

Ultra-low emission zone (ULEZ): Seven stations were situated outside central London but within the ULEZ. The ULEZ is designed to reduce air pollution by encouraging the use of cleaner vehicles. The stations in this zone help in understanding the effectiveness of both the ULEZ policy and the additional impact of COVID-19 restrictions on air quality.

Greater London (LEZ): Eight stations were located in greater London, outside the ultra-low emission zone (ULEZ). These areas typically have lower traffic density compared to central London but still experience significant vehicular movement. Analysing data from these stations provides insights into the broader regional impact of lockdowns beyond the more strictly regulated zones.

The selection of these 20 stations allows for a comprehensive analysis of air quality across different urban and suburban environments. By comparing data from central London, the ULEZ, and greater London, we can assess the spatial variability in the impacts of lockdown measures.

The percentage of missing values (averaged over all stations) for the whole study period was 6%, 15%, and 21% for NO_2_, PM_10_, and PM_2.5_, respectively. We implemented the multiple imputation by chained equation (MICE) method [29] for imputing missing values. The MICE method is a flexible and widely used method that allows for the imputation of missing data by generating multiple plausible datasets and combining the results to account for the uncertainty associated with the missing values. This approach helps to ensure that our analyses remain accurate and unbiased, despite the presence of incomplete data.

In addition, meteorological data, including ambient temperature and relative humidity, were extracted from London Heathrow airport.

## 3. Methodology

State-space intervention statistical modelling is an advanced technique used to analyse time series data, particularly when the objective is to understand the effects of specific interventions or events over time. This method is grounded in state-space models, which are characterized by their ability to handle complex temporal and spatial dependencies in the data. We have selected the state-space intervention model due to its ability to effectively handle time series data with abrupt changes, such as those caused by COVID-19 lockdowns. This method is particularly suited to our data’s characteristics, as it captures the temporal correlations and dynamic fluctuations in air pollution levels. Moreover, compared to other statistical techniques, such as VARMA, the state-space model offers advantages in accuracy and computational efficiency, making it a robust choice for analysing spatially distributed data [30].

State-space models consist of two main components: the observation equation and the state equation. The observation equation links the observed data to the unobserved state variables, while the state equation describes the evolution of these state variables over time. This framework is highly flexible, allowing for the incorporation of multiple sources of variation and the modelling of dynamic systems.

In the context of intervention analysis, state-space models are particularly powerful. The model can accommodate sudden changes or disruptions in the time series, such as lockdowns. By including intervention variables in the model, it is possible to quantify the impact of these interventions on the observed outcomes. For instance, in this study, state-space models can assess how lockdown measures influenced pollution levels, adjusting for other factors like meteorological conditions.

Moreover, state-space models can incorporate external covariates, making them suitable for complex real-world datasets. Their ability to capture both short-term fluctuations and long-term trends allowed us to disentangle the effects of interventions from underlying trends and seasonal patterns. This is achieved through the model’s latent state variables, which can represent various underlying processes driving the observed data.

In this study, the state-space intervention method’s application provides several benefits:

Flexibility: It can incorporate various types of data, including meteorological variables, which can affect air pollution levels.

Dynamic analysis: It accounts for changes over time and helps identify patterns and trends that simpler models might miss.

Multiple interventions: It can model the effects of multiple interventions, such as different phases of lockdowns, making it particularly suitable for studying the impacts of COVID-19 restrictions.

In the following, different parts of this model are described, and the complete model is presented at the end of this section.

### 3.1. COVID-19 Related Lockdowns

**Figure 2:**
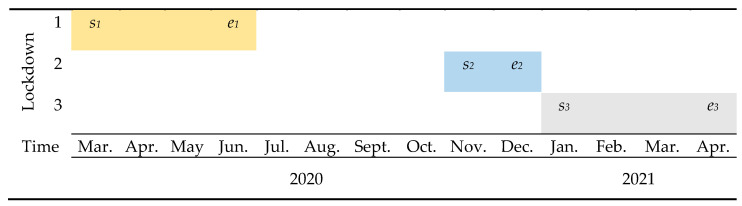
UK legal restrictions related to COVID-19.

The UK faced three major lockdowns in March 2020, November 2020, and January 2021, and each had a set of rules and restrictions. In lockdown 1, announced on 23 March 2020, people were asked to stay at home and just go outside for essential needs. These restrictions were relaxed on 23 June 2020. The second national lockdown was announced on 5th Nov. 2020 and people were asked to work from home again and non-essential businesses were closed. The second lockdown was eased on 15 December 2020. On 6 January 2021, England entered the third national lockdown, which was released step by step, up to 12 April 2021. Figure 2 indicates the timings of these lockdowns.

If *s_z_* (*z* ≥ 1) is the time at which legal restriction *z* was introduced, *e_z_* (*z* ≥ 1) is the time at which legal restriction *z* was rescinded, and *L_i_* represents the magnitude of the effect of legal restriction *z*, then the representation of the constant proportional effect at time *t* of a single legal restriction *z* that was in force during the interval (*s_z_*, *e_z_*] is as follows:

(1) $$L_{z}\left(t\right)=\left(\beta_{z}+\alpha_{z}t\right)\times \left(\Theta \left(t-s_{z}\right)\;\left[1-\Theta \left(t-e_{z}\right)\right]\right)$$

where Θ(*t*) is the Heaviside step function to represent the interval of each lockdown:

(2) $$\Theta \left(t\right)=\left\{\begin{array}{cc} 0 & \left(t\leq 0\right)\\ 1 & \left(t>0\right) \end{array}\right.$$

The first term in Equation (1), $\beta_{z}+\alpha_{z}t$, represents the changes related to lockdown *z* with a linear model (β is the constant of this model, and α is the slope). In fact, instead of using one parameter that applies consistently over the lockdown interval, we used a linear form. This approach allowed us to examine the dynamic impacts of each specific lockdown on air pollution, tracking changes from the start to the end of the lockdown interval.

### 3.2. Seasonal Effects

To accommodate the seasonal variation in pollutant concentrations over the 12 calendar months, the covariate vector δ*_m_* with coefficients $M_{m}$ was defined to represent the effects of month *m*. This covariate is defined as follows:

(3) $$\delta_{mt}=\left\{\begin{array}{l} 1\;\mathrm{if}\;\mathrm{time}\;t\;\mathrm{is}\;\mathrm{during}\;\mathrm{calendar}\;\mathrm{month}\;m\;\;\left(1\leq m\leq 12\right)\\ 0\;\mathrm{otherwise} \end{array}\right.$$

### 3.3. Process Matrix

The process matrix, B, represented the relationship of air pollution measurements at each monitoring station, *k* (1 ≤ *k* ≤ K), at time *t*, with the measurement at *t* − 1 (1 day lag) at the same station, as well as corresponding measurements at other stations. In fact, this matrix defined the spatio-serial relationship of pollutant concentrations as they developed over time and location (monitoring station). The diagonal elements of this matrix were the autoregressive coefficients with degree 1 (1 day lag) at each station; hence, they showed the concentration development over time. The off-diagonal elements were the first degree of autoregressive coefficients at other stations; therefore, they represent the relationship of concentrations over locations (monitoring stations).

### 3.4. State-Space Model

In the state-space model used for London air quality, the observed time series vector $y_{t}$ was a measurement of the atmospheric concentration of pollutants (observation vector) and the state time series vector $x_{t}$ was the corresponding vector of the atmospheric concentration of pollutants. Daily observations of ambient temperature and relative humidity were extracted from London Heathrow airport located in west London, provided by the National Centres for Environmental Information [31]. These meteorological measurements were applied with spatial uniformity over the study area. The meteorological variables including temperature, *T*, and humidity, *h*, were added to the model as covariates with coefficients γ and *χ*, respectively. Hence, the air quality state-space model was defined as follows:

(4) $$\begin{array}{l} y_{t}=x_{t}+\varepsilon_{t}\\ x_{t}=Bx_{t-1}+\sum_{z=1}^{3}L_{z}+\sum_{m=1}^{12}M_{m}\delta_{mt}+\gamma T_{t}+\chi h_{t}+w_{t} \end{array}$$

where $\varepsilon_{t}$ and $w_{t}$ are uncorrelated white noise representing the observation (process) error.

## 4. Results

### 4.1. Seasonal Effects (M) and Meteorological Factors

**Figure 3:**
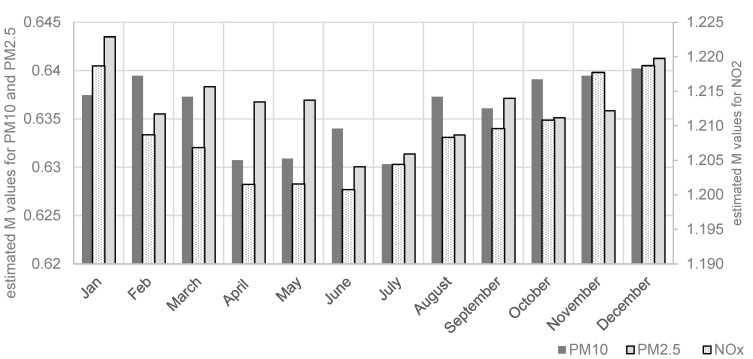
Estimated seasonal effects (M) for NO_x_, PM_10_, and PM_2.5_.

Parameter M represents the monthly effects on London’s air quality, which was supposed to remain unaffected by COVID-19 lockdowns. The parameter values are plotted in Figure 3 for NO_x_, PM_10_, and PM_2.5_. From these results, the variations of air pollution concentrations over calendar months are small, with a greater reduction from March to October.

For meteorological factors, we found a negative association between NO_2_, PM_2.5_, and PM_10_ and ambient temperature: for every 1 °C increase in temperature, NO_2_ levels decreased by approximately 1 µg/m^3^, while PM_2.5_ and PM_10_ concentrations decreased by around 1.5 µg/m^3^ each. In contrast, relative humidity showed a positive association with these pollutants. For every 10% increase in relative humidity, concentrations of NO_2_, PM_2.5_, and PM_10_ increased by approximately 1.5 µg/m^3^, 6 µg/m^3^, and 4 µg/m^3^, respectively.

### 4.2. COVID-19 Lockdowns Effects on London’s Air Pollution

We estimated the slope, α, and intercept, β, for lockdowns 1, 2, and 3 (L_1_, L_2_, and L_3_). Table 1 shows these results for NO_x_, PM_10_, and PM_2.5_, in central London (CL), the ultra-low emission zone (ULEZ), and greater London (GL), separately.

Parameter L_1_ had a negative intercept in all three zones of London for NO_x_, PM_10_, and PM_2.5_ (on average, −46.80, −5.42, and 5.02, respectively). This means lockdown 1 resulted in a sudden decrease in levels of air pollution; however, the estimated slope (*α*) for this lockdown was positive, which means that after the sudden decrease in the beginning of lockdown, air pollution gradually increased (with average slope = 5.44, 1.09 and 1.13 for NO_x_, PM_10_ and PM_2.5_, respectively) until the end of lockdown.

The effects of lockdown 2 on air pollution were different for NO_x_ compared to PM_10_ and PM_2.5_: while the intercept was negative for NO_x_ (−8.11), this value was positive for PM_10_ and PM_2.5_ (12.78 and 12.62, respectively). That means that at the beginning of lockdown 2, NO_x_ levels were reduced, but PM_10_ and PM_2.5_ levels were increased. However, the slope of changes was negative in all three pollutant types (average *α* = −11.94, −7.25, and −8.86, for NO_x_, PM_10_, and PM_2.5_, respectively), which means the levels of all three pollutant types were lower at the end of this lockdown, compared to the start of this lockdown.

Trends during lockdown 3 were similar to lockdown 1: there was a negative intercept and positive slope for all pollutant types. For NO_x_, the intercept in lockdown 3 was 30% lower than lockdown 1, and the slope was 2.5 times higher in L_3_ compared to L_1_. That means that during the third lockdown, NO_x_ levels were reduced at the start (but not as much as lockdown 1), but then increased with a greater slope compared to lockdown 1. For PM_10_ and PM_2.5_, the slopes in L_3_ were nine times higher than the corresponding value in L_1_. The intercept for these pollutants in L_3_ were also three times higher than in L_1_. Again, this means that the changes in the PM_10_ and PM_2.5_ levels during the third lockdown were more rapid than the first lockdown.

These results highlight the varying impacts of different lockdown phases on air pollution levels in London. The initial reductions in pollution during each lockdown demonstrate the effectiveness of reduced human activity in lowering emissions. However, the subsequent increases, particularly during L_1_ and L_3_, suggest that other factors, such as changes in industrial activity or increased localized pollution sources, might have influenced these trends.

Lockdown 2’s unique pattern, with initial increases in PM_10_ and PM_2.5_, could be attributed to specific local activities. One reason could be attributed to the chemical reactions in the atmosphere that can lead to the formation of secondary pollutants. For instance, NO_x_ can react with other compounds to form secondary particulate matter (PM_2.5_). During lockdown 2, changes in precursor emissions could have favoured the formation of secondary PM_2.5_, leading to higher levels despite a decrease in NO_x_. Furthermore, the increased use of household products, cleaning agents, and other VOC-emitting activities during the lockdown could contribute to the formation of secondary organic aerosols, a component of PM_2.5_.

**Figure 4:**
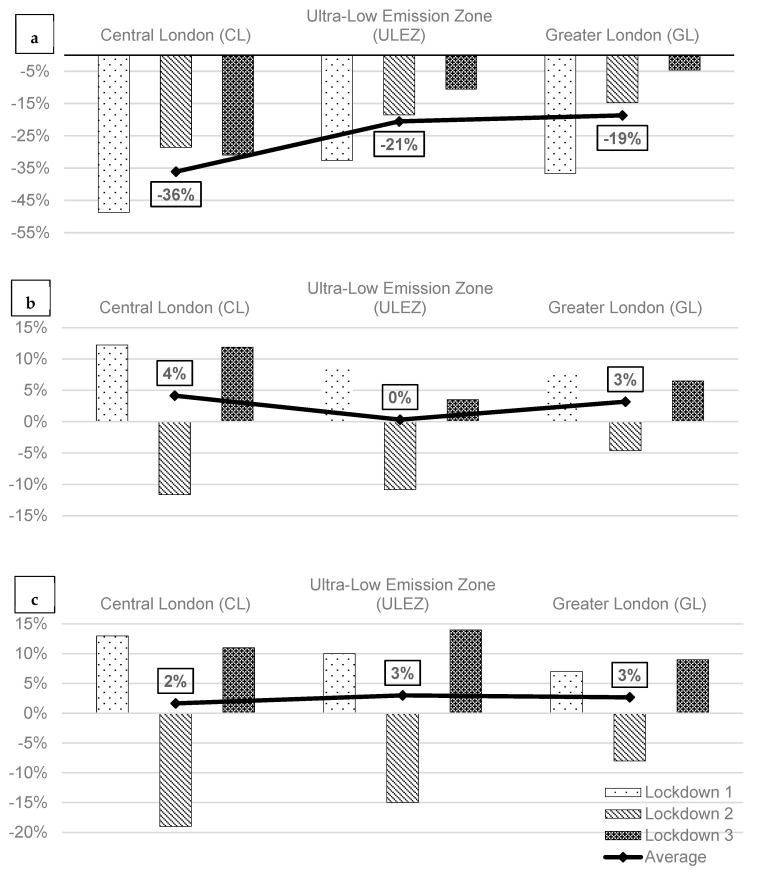
Percentage of changes in air pollution levels during lockdown 1, 2, and 3 for 3 zones of London (CL, ULEZ, and GL), (**a**) NO_x_, (**b**) PM_10_, and (**c**) PM_2.5_.

The estimated intercept and slope of changes during lockdowns can be converted to a percentage of changes in the levels of air pollution during each lockdown. Figure 4 shows changes in percentages for NO_x_, PM_10_, and PM_2.5_, in central London, the ULEZ, and greater London during all three lockdowns.

The percentage changes in air pollution levels across the three zones provide further insights. Central London (CL) experienced the most significant reductions in NO_x_, with an average decrease of 36% across the three lockdowns. This reflects the high initial traffic density and the substantial impact of reduced vehicular movement in the city centre. In contrast, PM_10_ and PM_2.5_ levels in CL showed slight increases of 4% and 1%, respectively, likely due to sources such as construction activities and secondary formation processes that were less affected by the lockdowns.

In the ULEZ, NO_x_ levels decreased by 21%, while the increases in PM_10_ and PM_2.5_ were less pronounced compared to central London. This suggests that the existing emission controls in the ULEZ mitigated some of the potential increases in particulate matter. The effectiveness of stringent vehicle emission policies is evident, even during the unusual circumstances of the lockdowns.

Greater London (GL) showed the smallest reduction in NO_x_ levels (19%) and modest increases in PM_10_ and PM_2.5_. The suburban and less densely populated nature of this area likely contributed to these more subdued changes, highlighting the importance of targeted interventions in areas with higher baseline pollution levels.

These findings have important implications for future air quality management and policy-making processes. The effectiveness of the ULEZ in mitigating increases in particulate matter highlights the value of stringent emission controls. Additionally, the results suggest that policies promoting remote work, cleaner vehicle technologies, and better management of construction activities could have lasting positive impacts on urban air quality. Overall, the varying impacts of the three lockdowns on air pollution levels underscore the complex interplay between different sources of pollution and the influence of external factors such as human behaviour. The significant reductions in NO_x_ across all zones demonstrate the immediate benefits of reduced vehicular and industrial activities, while the increases in particulate matter levels in some areas suggest the need for comprehensive strategies addressing all pollution sources.

The results of this study, which highlight the impact of COVID-19 lockdowns on air pollution levels in London, are consistent with findings from several other countries, albeit with some variations attributable to regional differences in industrial activities, urban density, and local environmental policies.

In China, several studies reported significant reductions in NOx, PM2.5, and PM10 during lockdown periods. For instance, Zhao et al. [11] observed a reduction in NO2 by up to 60% in major cities, which is more pronounced compared to the reduction in NOx observed in central London during the first lockdown. The more stringent lockdown measures and higher initial pollution levels in China likely contributed to these more significant reductions. Similarly, in India, Mahato et al. [14] reported a substantial decline in NO2 levels (up to 70%) in Delhi during the lockdown, which is again higher than the reductions seen in London. However, similar to London, PM2.5 levels in some regions did not decline as significantly, and in some cases, even increased, which is attributed to continued biomass burning and other non-vehicular sources.

In contrast, in central Europe, Polednik [13] reported more modest reductions in NOx (around 30%) and less significant changes in particulate matter levels during the lockdowns. This is closer to the changes observed in London, particularly in the ultra-low emission zone (ULEZ) and greater London areas, where the reductions were less drastic due to existing emission controls and the less severe lockdown measures compared to those in China and India. In Brazil, particularly in the state of São Paulo, Cirqueira et al. [12] found a heterogeneous impact on air quality, with some areas showing reductions in NOx similar to those in London, while others exhibited little to no change. The variability was linked to differing levels of adherence to lockdown measures and the continued operation of industrial activities in some areas. Further supporting these findings, Begou et al. [32] investigated the impact of lockdowns on air quality in western Macedonia, Greece. They observed variability in pollutant concentrations, with NO2 reductions comparable to those seen in London. However, like in other regions, the changes in particulate matter were less consistent, emphasizing the influence of local meteorological conditions and varying sources of pollution.

While this study provides valuable insights into the impacts of lockdowns on air quality, there are several limitations to consider. First, the analysis relied on data from 20 monitoring stations, which, while comprehensive, may not fully capture the micro-level variations in air quality across different urban environments. Additionally, the model assumed uniform meteorological conditions across London, which may oversimplify the complex interactions between local weather patterns and pollutant dispersion. The study also primarily focused on the immediate effects of lockdowns, potentially overlooking longer-term changes in air pollution sources, such as shifts in traffic patterns post-lockdown. Moreover, the use of a state-space model, though advanced, might not account for all confounding factors, such as unmeasured economic activities or non-traffic-related pollution sources, that could have influenced the observed outcomes.

For future research, expanding the spatial scope of the study to include more monitoring stations and incorporating high-resolution meteorological data could provide a more detailed understanding of pollution dynamics. Additionally, future work could explore the long-term impacts of altered traffic patterns post-COVID-19 and assess the effectiveness of different public health policies aimed at reducing air pollution. Integrating more sophisticated models that can account for nonlinear relationships and additional variables, such as socio-economic factors or public transport usage, could also improve the robustness of the findings. Finally, comparative studies across different cities with similar lockdown measures could help generalise the results and provide broader policy implications.

## 5. Conclusions

This study employs a state-space intervention model to investigate the impact of the COVID-19 lockdowns on air pollution levels in London, focusing on NOx, PM_10_, and PM_2.5_. Our findings reveal that the first lockdown had the most significant impact, with substantial reductions in NOx levels across central London, the ULEZ, and greater London. However, this lockdown also saw increases in PM_10_, and PM_2.5_, likely due to higher residential energy consumption. The subsequent lockdowns, while still effective in reducing NOx, showed less pronounced effects and more variable impacts on particulate matter concentrations.

The state-space model’s ability to account for seasonal variations, ambient temperature, and relative humidity allowed for a nuanced understanding of these dynamics. The results underscore the importance of targeted interventions to address specific pollution sources, particularly those related to road transport. They also highlight the complex interplay between different pollutants and the varying impacts of lockdown measures over time.

This research contributes valuable insights into the effectiveness of lockdown measures on urban air quality and suggests that similar strategies, such as promoting remote work and cleaner vehicle technologies, could be effective in managing air pollution in non-crisis periods. The application of state-space modelling in this context demonstrates its utility in environmental health research, providing a robust framework for analysing the temporal and spatial impacts of significant interventions.

## Figures and Tables

**Table 1:** Intercept (β) and slope (α) of parameters L_1_, L_2_, and L_3_, for NO_x_, PM_10_, and PM_2.5_, in central London (CL), the ultra-low emission zone (ULEZ), and greater London (GL).

Pollutant	Zone	Lockdown 1 (L_1_)		Lockdown 2 (L_2_)		Lockdown 3 (L_3_)	
		*β*_1_	*α*_1_	*β*_2_	*α*_2_	*β*_3_	*α*_3_
NO_x_	CL	−48.11	5.36	−19.12	−4.21	−48.23	17.14
	LEZ	−43.51	5.39	8.21	−30.2	−21.91	9.14
	GL	−48.78	5.44	−13.41	−1.42	−26.5	14.47
	average	−46.80	5.40	−8.11	−11.94	−32.21	13.58
PM_10_	CL	−6.22	1.16	15.15	−8.44	−16.34	8.90
	LEZ	−4.13	0.80	13.63	−7.69	−15.37	9.56
	GL	−5.92	1.32	9.49	−5.61	−18.94	11.24
	average	−5.42	1.09	12.76	−7.25	−16.88	9.90
PM_2.5_	CL	−4.35	1.20	12.89	−13.15	−23.70	13.28
	LEZ	−6.20	1.48	15.84	−8.21	−6.84	5.94
	GL	−4.51	0.72	9.13	−4.56		
	average	−5.02	1.13	12.62	−8.64	−15.27	9.61

## Data Availability

The original data presented in the study are openly available in Londonair.org.uk, accessed on 21 August 2024.
